# Elucidation of the Pathogenesis of Autoinflammatory Diseases Using iPS Cells

**DOI:** 10.3390/children8020094

**Published:** 2021-02-01

**Authors:** Megumu K. Saito

**Affiliations:** Department of Clinical Application, Center for iPS Cell Research and Application, Kyoto University, Kyoto 6068507, Japan; msaito@cira.kyoto-u.ac.jp

**Keywords:** iPS cells, autoinflammatory diseases, macrophages, somatic mosaicism, genome editing

## Abstract

Autoinflammatory diseases are a disease entity caused by the dysregulation of innate immune cells. Typical autoinflammatory diseases are monogenic disorders and often very rare. As a result, there is a relative lack of understanding of the pathogenesis, poor diagnosis and little available treatment. Induced pluripotent stem (iPS) cells are a new technology being applied to in vitro disease modeling. These models are especially useful for the analysis of rare and intractable diseases including autoinflammatory diseases. In this review, I will provide a general overview of iPS cell models for autoinflammatory diseases and a brief description of the results obtained from individual reports.

## 1. Introduction

Autoinflammatory diseases are a disease entity first proposed in 1999 as a group of diseases in which inflammatory findings show no apparent triggers and no evidence of existing autoantibodies or autoreactive T cells [1]. Autoinflammatory diseases are mainly caused by abnormalities in innate immunity, which contrasts them with autoimmune diseases, which are caused by abnormalities in acquired immunity. The original definition of autoinflammatory diseases made them consistent with congenital monogenic disorders, which are traditionally referred to as hereditary periodic fever syndromes. However, the concept of autoinflammatory diseases has since expanded to include multifactorial genetic diseases such as PFAPA syndrome, Behcet’s disease, adult Still’s disease, and gout [2,3].

Because autoinflammatory diseases are a relatively new disease concept and because they are mainly composed of rare genetic diseases, they are not widely recognized by physicians or scientists. Therefore, there remains little in terms of diagnostic and therapeutic techniques and understanding of the pathogenic mechanisms. In this regard, research using induced pluripotent stem (iPS) cells for the analysis of autoinflammatory diseases has grown. In this paper, I present examples of iPS cell applications for the pathological analysis of autoinflammatory diseases and discuss future perspectives.

## 2. Disease Modeling Using iPS Cells

iPS cells are pluripotent stem cells established by Shinya Yamanaka and colleagues [4,5]. Because of their pluripotency, pluripotent stem cells, such as embryonic stem cells (ES cells) and iPS cells, can differentiate into all types of cells and tissues that make up an individual. They also have the ability to self-renew. One of the unique features of iPS cells is that they can be established from somatic cells. Therefore, it is possible to establish iPS cells from blood cells or skin fibroblasts from a patient with hereditary diseases. Importantly, these iPS cells share the genetic background of the patient. The resulting iPS cells can then be differentiated into the cell types responsible for the disease pathogenesis, and the resulting phenotypes can be analyzed for in vitro disease modeling. Indeed, iPS cell-based disease models have been effectively used for the diagnosis, pathological analysis, and development of therapies for various intractable pediatric diseases and have already become indispensable tools for disease research [6,7,8].

For these reasons, iPS cell models are expected to accelerate the understanding and treatment of autoinflammatory diseases, especially those caused by single gene mutations. From patient iPS cells, researchers can prepare the cell types, such as neutrophils, monocytes, and macrophages, that show the disease phenotype.

## 3. Differentiation of iPS Cells into Innate Immune Cells

There already exist many differentiation systems to obtain the above innate immune cells from iPS cells [9,10,11,12,13,14,15]. Macrophages derived from pluripotent stem cells are thought to mimic primitive macrophages derived from primitive hematopoiesis [16]. These macrophages can be further differentiated into tissue-resident macrophages, which can be used as a model for local inflammatory responses. In the differentiation of hematopoietic cells from human pluripotent stem cells, hematopoietic progenitor cells need to be induced first. The combination of cytokines in this differentiation protocol is optimized for each cell type. For example, stem cell factor, IL-3, and macrophage colony-stimulating factor are used to induce monocytic cells [15], which can then be differentiated into functional dendritic cells and macrophages. Since the induction of each differentiated cell type requires significant amounts of human and financial resources, it is important to clarify the target cell type and construct an experimental plan by predicting the phenotype to be studied.

## 4. Pathological Analysis of Autoinflammatory Diseases Using iPS Cells

Table 1 shows examples of autoinflammatory diseases modeled with iPS cells. Although some articles only reported the establishment of iPS cells from patients with autoinflammatory diseases, many others further describe differentiated myeloid cells and corresponding pathology assays [17,18,19,20,21,22]. In particular, recent advances in differentiation techniques have led to the functional analysis of trophoblast-like cells [23] and chondrocytes [24]. In most cases, the number of patients is only one or two, which is extremely small. Thus, attempts have been made to create isogenic iPS cell clone pairs by knocking in the genetic mutation or by repairing the patient’s genetic mutation using genome editing technology. Since monogenic autoinflammatory diseases are rare, meaning patient subjects for iPS cell-based study is rare, isogenic clones are often useful to evaluate the disease-associated phenotypes. In the following sections, I pick up several studies and discuss their details and significance.

## 5. Somatic Mosaicism of Chronic Infantile Neurocutaneous Arthropathy (CINCA) Syndrome

Chronic infantile neurocutaneous arthropathy (CINCA, MIM; 607115) syndrome, also known as neonatal onset multisystem inflammatory disease (NOMID), is an autoinflammatory syndrome with a triad of non-pruritic urticarial rash, meningitis, and joint lesions that develops in the neonatal period [30] and is the most severe form of the autoinflammatory spectrum called cryopyrin-associated periodic fever syndrome. Patients carry a heterozygous gain-of-function mutation in the *NLRP3* gene and present systemic inflammation due to excessive IL-1β production caused by hyperactivation of the inflammasome [31,32]. The genetics of CINCA syndrome are characterized by the presence of *NLRP3* mutations as low frequency somatic mosaicisms rather than constitutive in about 30–40% of patients [33,34,35]. However, even if the percentage of *NLRP3* mutant cells in somatic cells is about 10%, the phenotype is comparable to that of patients with mutations in all cells in the body, raising the question of whether only these low-frequency *NLRP3* mutant-positive cells are responsible for the disease or whether cells other than those with *NLRP3* mutations also contribute to the pathogenesis.

One of the characteristics of iPS cells is that the source of each iPS cell clone is derived from a single somatic cell [36]. Therefore, if an individual has a certain genetic mutation as a somatic mosaicism and multiple iPS clones are established from the patient, mutant and wild-type iPS cells can be obtained. In other words, cells with each genotype can be isolated from individuals with a mixture of cells with and without the genetic mutation. Taking advantage of this feature, *NLRP3*-mutant and wild-type iPS cells were established from CINCA syndrome patients with a somatic NLRP3 mutation [19]. When these iPS cells were differentiated into macrophages and their phenotypes were compared, only the *NLRP3*-mutant macrophages produced a large amount of IL-1β. Interestingly, when mutant macrophages were mixed with wild-type macrophages to create a pseudo-mosaic state and stimulated, IL-1β production was enhanced compared to mutant macrophages alone. In other words, in the somatic cell mosaicism, mutant cells mainly produce IL-1β, but wild-type cells also contribute to inflammation in some way. Thus, iPS cells can be used for detailed analysis of the unique pathologies associated with somatic cell mosaicisms.

## 6. Analysis of Cartilage Lesions in CINCA Syndrome

In CINCA syndrome, contractures with periarticular bone hypertrophy are often observed, and the pathophysiology has been thought to be the hyperplasia of growing cartilage [37]. However, the mechanism of this hyperplasia has been unclear, and it was not known whether IL-1β-dependent inflammation contributed to it. In a study attempting to reproduce the cartilage pathology of CINCA syndrome, iPS cells from patients with the aforementioned mosaic CINCA syndrome were differentiated into chondrocytes [24]. The cartilage produced from *NLRP3* mutant clones derived from the same patient was larger than that from wild-type iPS cells, and calcification occurred in a disordered manner. A detailed examination of the mechanism of cartilage hypertrophy suggested that the mechanism lies behind the increased expression of the *SOX9* gene due to enhancement of the cAMP-cAMP responsive element binding protein (CREB) pathway. These results are important for understanding cartilage lesions in CINCA syndrome and for developing novel therapies, and provide an example of how iPS cells can be used to analyze cartilage lesions, which was previously difficult to do.

## 7. Diagnosis of Somatic NLRC4 Mosaicism in CINCA Syndrome

As mentioned above, in CINCA syndrome, about 90% of patients have constitutive or somatic mosaic mutations in *NLRP3*. However, the remaining 10% of patients with clinical symptoms of CINCA syndrome do not have mutations in the *NLRP3* gene [35]. Since most of these patients respond to anti-IL-1 therapy, it had been assumed that there was a common mechanism of the disease development, but the causative gene was unknown. Therefore, we established iPS cells from a patient with CINCA syndrome who did not have the *NLRP3* mutation, induced them to differentiate into macrophages, and confirmed the production of IL-1β [28]. Each clone of iPS cells showed different reactivity, and the phenotype was divided into “normal” clones that secreted IL-1β normally and “pathological” clones that secreted IL-1β excessively. Since each iPS cell clone was derived from a single somatic cell as I mentioned, we considered this patient to be a somatic mosaicism of an unknown disease-related gene mutation. Therefore, we attempted to detect specific mutations in the diseased clones using whole exome sequencing. As a result, mutations in the *NLRC4* gene were commonly detected in the diseased clones. When we knocked out the *NLRC4* gene in the iPS cell clones with the mutated *NLRC4* gene, the production of IL-1β decreased and the clone recovered to the normal phenotype. These results proved that the somatic mosaicism of the *NLRC4* gene mutation is the genetic background of this CINCA syndrome patient. To date, no other case of somatic mosaicism of *NLRC4* has been reported, nor has *NLRC4* been reported as the causative gene of CINCA syndrome. Thus, this study showed that iPS cell technology can play an essential role to accurately diagnose extremely rare cases and that dissection of iPS cell-derived phenotypes can be useful in diagnosing sporadic patients for whom the causative gene is difficult to identify.

## 8. Involvement of IFN-γ in the Development of Autoinflammation in Blau Syndrome

Blau syndrome (MIM; 186580) is a disease caused by a heterozygous gain-of-function mutation in the *NOD2* gene, which leads to granulomatous lesions in the skin, joints, and eyes in childhood and severe complications such as blindness and joint contractures [38,39,40]. In Blau syndrome, persistent tissue autoinflammation is observed. However, although several hypotheses for the mechanism of the inflammation have been proposed, little have been experimentally proven. In addition, studies using cultured cell lines and mouse models have not sufficiently reproduced the disease-related phenotype [41]. Therefore, we established iPS cells from patients with a *NOD2* mutation and isogenic iPS cells in which the mutation was repaired [21]. In addition, we generated iPS cells with the same *NOD2* mutation knocked into control iPS cells to create two pairs of iPS cell clones that are genetically homogeneous except for the difference in the *NOD2* genotype. When these iPS cells were induced to differentiate into macrophages and their cytokine production capacity was compared, it was found that IFN-γ-dependent activation of the NF-κB pathway and production of pro-inflammatory cytokines were induced in the *NOD2* mutant clones. Considering that granulomas in Blau syndrome patients are IFN-γ positive [42] and that some patients develop the disease after BCG vaccination [43,44,45], which induces IFN-γ production, our findings suggest that IFN-γ may trigger autoinflammation in Blau syndrome patients.

## 9. Construction of a Pathological Model for Nakajo-Nishimura Syndrome

Nakajo–Nishimura syndrome (NNS), also known as proteasome-associated autoinflammatory syndrome 1 (PRAAS1, OMIM; 256040) is a disease characterized by chronic inflammation and lipomuscular atrophy caused by homozygous loss-of-function mutations in the *PSMB8* gene encoding β5i, a component of the immunoproteasome [46,47]. Recently, Janus kinase (JAK) inhibitors have been reported to be effective for the treatment of diseases with *PSMB8* mutations [48]. However, the precise mechanism of the autoinflammation remain unknown. We generated iPS cells from an NNS patient and repaired clones of the *PSMB8* mutation in order to develop a pathological model of NNS [22]. In parallel, we transduced control human ES cells with the *PSMB8* mutation gene and generated two pairs of pluripotent stem cells that are genetically uniform except for the difference in *PSMB8* genotype, as in the case of Blau syndrome. After differentiation of these pluripotent stem cells into monocytic cells, they were stimulated with IFN-γ and TNF-α, both of which are immunoproteasome-inducing stimuli. The results showed that the function of the immunoproteasome was significantly reduced in *PSMB8* mutant monocytes compared to normal monocytes, while the secretion of pro-inflammatory cytokines and chemokines was enhanced. Interestingly, mitochondria-derived reactive oxygen species production was enhanced in the mutant cells, and the addition of antioxidants suppressed the production of pro-inflammatory cytokines and chemokines. Thus, oxidative stress was shown to be involved in the enhancement of the inflammatory response by *PSMB8* mutation. This study demonstrates the usefulness of patient iPS cells for modeling proteasome-associated autoinflammatory diseases.

## 10. Inflammatory Bowel Disease Caused by Loss of IL-10 Signaling

IL-10 is one of the most important cytokines for the maintenance of intestinal homeostasis and regulates inflammation by inhibiting macrophage activation [49,50,51]. It has been reported that lost function of the IL-10 signaling pathway, i.e., loss-of-function mutations in IL-10 and IL-10 receptors, causes severe early-onset inflammatory bowel disease (IBD) [52,53,54,55]. Therefore, to investigate the effect of IL-10 deficiency on macrophage function, iPS cells were established from patients with homozygous loss-of-function mutations in IL-10 receptor β (IL-10RB) and differentiated into macrophages [17]. In that report, IL-10RB-/- patient iPS cell-derived macrophages were found to be deficient in the IL-10 signaling pathway, and suppression of pro-inflammatory cytokine secretion was not observed upon simultaneous IL-10 and LPS stimulation. Interestingly, the prostaglandin E2 (PGE2)-associated pathway was enhanced in IL-10RB-/- patient iPS cell-derived macrophages, but the bactericidal activity was reduced. Furthermore, the combined inhibition of PGE2 synthesis and receptor binding improved the bactericidal activity. These results indicate that there is crosstalk between the IL-10 and PGE2 pathways and that IL-10RB-deficient macrophages have two negative aspects that contribute to inflammation: overactivation and reduced cell killing capacity. Thus, the use of iPS cell-derived macrophages revealed an unexpected crosstalk between inflammatory pathways. This is a very interesting result and useful for considering inflammation control strategies. In the future, it may be possible to co-culture intestinal epithelial cells and macrophages in pluripotent stem cell-derived intestinal organoids [56,57,58,59], which would be useful for evaluating the inflammatory crosstalk responsible for the development of IBD.

## 11. Abnormal Trophoblast Differentiation in Familial Recurrent Hydatidiform Moles

Familial recurrent hydatidiform moles (hydatidiform mole, recurrent 1; HYDM1; MIM 231090) is an inherited disorder that causes recurrent complete hydatidiform moles [60]. It is characterized by the presence of normal diploid mole tissue and abnormal DNA methylation. The cause of this disease is known to be maternal biallelic *NLRP7* mutations [25,61,62]. *NLRP7* is an inflammasome protein, but the mechanism by which *NLRP7* dysfunctions lead to complete hydatidiform moles is unknown. The *NLRP7* gene does not have a mouse orthologue, which makes it difficult to construct animal models. Since the origin of hydatidiform moles is trophoblasts, it is necessary to induce this cell type from iPS cells. In a report on the establishment of iPS cells from a case of HYDM1 with a compound heterozygous mutation, phenotypic analysis was attempted using a system to induce trophoblasts in vitro [23]. The results showed that pluripotency factors were downregulated early in *NLRP7*-deficient iPS cells and trophoblast-related markers were upregulated. Furthermore, these phenotypes were dependent on BMP4 signaling, and inhibition of the BMP pathway corrected the excessive trophoblast differentiation of the patient-derived iPS cells. Although functional analysis was not performed in detail in this study, gene expression profiling analysis led to interesting results and insights. For example, the model provides a new method for analyzing human placental diseases. In addition, *NLRP7*, an inflammasome-related molecule, was shown to be an important regulator of cell fate during human development. Autoinflammatory diseases show a wide variety of inflammatory lesions, but there are also lesions that do not have inflammatory cell infiltration. For example, the cartilage lesions of CINCA syndrome described above are also non-inflammatory lesions. Detailed analysis of lesion tissues using iPS cells may be useful for the analysis of such non-inflammatory lesions.

## 12. Conclusions

As illustrated in this review, iPS cell-based analysis is useful for the diagnostic and pathological analysis of autoinflammatory diseases. However, there may be cases and diseases where the correct cell to reproduce the phenotype in vitro using iPS cells has not been identified. In these cases, careful selection of the desired cell type and optimization the differentiation and analysis protocols are needed. As seen in the case of Blau syndrome, it is also important to select the appropriate stimulus to obtain the phenotype associated with autoinflammation. Another problem that is often faced in the study of autoinflammatory diseases is the small number of cases. When there are only one or two cases for which iPS cells can be established, it is often difficult to obtain statistically reliable quantitative phenotypes, especially if the control clones are chosen at random. In these cases, the generation of isogenic counterparts by genome editing technology allows us to more reliably evaluate the phenotypic impact of the mutation.

As mentioned in the introduction, the concept of autoinflammation is continuously expanding. Indeed, it has recently been reported that Down syndrome also has a spectrum of autoinflammation, since multiple interferon-related molecules are encoded on chromosome 21 [63]. There are several studies on Down syndrome using iPS cells, with a particular focus on hematopoietic abnormalities [64,65,66,67,68,69,70] and neurological symptoms [71,72,73,74,75]. Similar research on autoinflammation in Down syndrome may also be possible using iPS cells. Such research will benefit from technologies already developed to manipulate chromosomes in iPS cells [65,66,70].

In autoinflammatory diseases, it is important to analyze the functions of not only blood cells but also cells in various inflammatory tissues, such as the musculoskeletal system and vascular endothelial cells. Technology for the in vitro differentiation of iPS cells into various cell types or tissues has progressed considerably. Therefore, it is possible to gain a comprehensive understanding of autoinflammatory diseases by differentiating iPS cells into these other cell types. In addition to understanding autoinflammatory diseases better, iPS cell models are expected to lead to the development of new therapeutic methods.

## Figures and Tables

**Table 1:** Induced pluripotent stem cells (iPSCs) established from autoinflammatory disease patients. Responsible genes in the Infevers database [25] were searched on Pubmed.

Gene	Disease	Target Cell Type	Case Number	Reference
IL-10RA	Inflammatory bowel disease- 28	Not described	1	[26]
IL-10RB	Inflammatory bowel disease- 25	Macrophages	1	[17]
MEFV	Familial Mediterranean fever	Not described	1	[27]
MEFV	Familial Mediterranean fever	Myeloid cell lines	4	[18]
MEFV	Familial Mediterranean fever	Macrophages	1	[16]
NLRC4	CINCA syndrome	Myeloid cell lines	1	[28]
NLRP7	Recurrent hydatidiform moles	Trophoblast like cells	1	[23]
NLRP3	CINCA syndrome	Macrophages	2	[19]
NLRP3	CINCA syndrome	Chondrocytes	2	[24]
NLRP3	CINCA syndrome	Myeloid cell lines	1	[20]
NOD2	Blau syndrome	Myeloid cell lines	1 (+1 isogenic line)	[21]
PSMB8	Nakajo-Nishimura syndrome	Myeloid cell lines	1 (+1 isogenic line)	[22]
OTULIN	OTULIN-related autoinflammatory syndrome	Not described	1	[29]
